# Myocardin/microRNA-30a/Beclin1 signaling controls the phenotypic modulation of vascular smooth muscle cells by regulating autophagy

**DOI:** 10.1038/s41419-022-04588-0

**Published:** 2022-02-08

**Authors:** Danyang Shi, Jinhua Ding, Shouqiang Xie, Lei Huang, Hongmin Zhang, Xiaojie Chen, Xuejun Ren, Sa Zhou, Hongpeng He, Wenjian Ma, Tongcun Zhang, Nan Wang

**Affiliations:** 1 College of Biotechnology, Tianjin University of Science and Technology, Tianjin, China; 2 Key Laboratory of Industrial Fermentation Microbiology, Ministry of Education and Tianjin, Tianjin, China; 3 Department of Cardiology, Beijing Anzhen Hospital Affiliated to Capital Medical University, Beijing, China

**Keywords:** Autophagy, Cell growth, miRNAs

## Abstract

Upon vascular injury, vascular smooth muscle cells (VSMCs) change from a contractile phenotype to a synthetic phenotype, thereby leading to atherogenesis and arterial restenosis. Myocardin (MYOCD) is essential for maintaining the contractile phenotype of VSMCs. Deletion of MYOCD in VSMCs triggers autophagy. However, the molecular mechanism underlying the effect of MYOCD on autophagy is not clear. In this study, knockdown of MYOCD in human aortic VSMCs (HA-VSMCs) triggered autophagy and diminished the expression of SMC contractile proteins. Inhibition of autophagy in MYOCD-knockdown cells restored the expression of contractile proteins. MYOCD activated the transcription of miR-30a by binding to the CArG box present in its promoter, as confirmed by luciferase reporter and chromatin immune coprecipitation assays, while miR-30a decreased the expression of autophagy protein-6 (ATG6, also known as beclin1) by targeting its 3′UTR. Restoring the expression of miR-30a in MYOCD-knockdown cells upregulated the levels of contractile proteins. Treatment of VSMCs with platelet-derived growth factor type BB (PDGF-BB) resulted in the transformation of VSMCs to a proliferative phenotype. A low level of miR-30a was observed in PDGF-BB-treated HA-VSMCs, and re-expression of miR-30a led to a decrease in proliferative marker expression. Furthermore, using a wire injury mouse model, we found that miR-30a expression was significantly downregulated in the arterial tissues of mice and that restoration of miR-30a expression at the injured site abolished neointimal formation. Herein, MYOCD could inhibit autophagy by activating the transcription of miR-30a and that miR-30a-mediated autophagy defects could inhibit intimal hyperplasia in a carotid arterial injury model.

## Introduction

The switching of vascular smooth muscle cells (VSMCs) from a contractile, quiescent or differentiation phenotype to an adverse, synthetic, proliferative or dedifferentiation phenotype plays an important role in the pathogenesis of various vascular proliferative diseases, including atherosclerosis, restenosis, and aneurysm. Inappropriate VSMC activation and proliferation are critical causes of neointima formation, stenosis and obstructions of vessels. The proliferative phenotype of VSMCs is characterized by proliferation and migration, as well as decreased levels of contractile marker proteins such as alpha smooth muscle actin (ACTA2, alpha-SMA), smooth muscle 22 alpha (SM22α) and smooth muscle myosin heavy chain (SMMHC). Understanding the potential mechanisms of VSMC phenotypic switching provides a new therapeutic strategy for the treatment of vascular proliferative diseases.

Myocardin (MYOCD) is expressed specifically in cardiac myocytes and VSMCs and plays a critical role in cardiovascular development [1]. It is a serum response factor (SRF)-dependent cofactor and can activate the expression of smooth muscle-specific genes by binding to SRFs on CArG box-containing target genes [2]. During postnatal development, MYOCD is essential for maintaining homeostasis in the vascular system and visceral tissues. MYOCD expression represents a contractile and differentiated SMC phenotype. Deletion of MYOCD, however, represents a synthetic and dedifferentiated phenotype, a hallmark of atherosclerosis [3]. Using tamoxifen-treated *SMMHC-Cre*^*ERT2*^*/Myocd*^*F/F*^ conditional mutant mice, Jianhe Huang et al. confirmed that loss of MYOCD markedly decreased the expression of SMC contractile proteins and induced cell autonomous autophagy [4]. This phenomenon suggests a new feature of MYOCD in regulating autophagy. However, it is not clear why the loss of MYOCD causes autophagy activation in SMCs.

Increasing evidence has confirmed the critical role of autophagy in the smooth muscle cell phenotype switch. Autophagy is one of the key degradation systems devoted to degrading and recycling misfolded proteins and damaged or aged organelles. Autophagy activation can be observed in VSMCs in vascular diseases, including atherosclerosis [5], hypertension [6] and dissecting aortic aneurysms (AAs) [7]. Various stimuli and stressors, such as reactive oxygen and lipid species [8], proinflammatory cytokines [9, 10] and shear stress [11], have been shown to induce autophagy in VSMCs. It has been reported that various stimuli, including secreted protein sonic hedgehog [12], tumor necrosis factor-α (TNF-α) [9], platelet-derived growth factor type BB (PDGF-BB) [10], and nicotine [13], inhibit the expression of key VSMC contractile genes and induce dedifferentiation of VSMCs, as well as the activation of autophagy. Activation of autophagy induced by PDGF-BB results in the degradation of contractile proteins, leading to the dedifferentiation phenotype of VSMCs [10]. However, it is still unclear how activation of autophagy regulates the expression of synthetic proteins in VSMCs.

MicroRNAs (miRNAs) are a class of endogenous, 22–24 nucleotide noncoding RNAs that function as posttranscriptional regulators of gene expression. MiRNAs have been found to participate in a variety of biological processes ranging from critical physiological functions to the pathophysiology of many diseases. Recent studies have confirmed the important role of miRNAs in the posttranslational orchestration of autophagy-related genes. MiRNA-30a has been reported to inhibit autophagy by directly targeting beclin 1 in various types of cells [14–16]. However, the role of miRNA-30a in the phenotypic switch of vascular smooth muscle cells is still unknown.

In this study, we revealed a new mechanism by which MYOCD regulated the expression of smooth muscle contractile genes. We found that MYOCD could maintain the expression of SMC contractile proteins by upregulating miRNA-30a-5p and inhibiting VSMC autophagy. We have also demonstrated for the first time that miRNA-30a-5p plays an important role in regulating the phenotypic switch of VSMCs.

## Results

### Knockdown of MYOCD triggered autophagy in HA-VSMCs

**Fig. 1:**
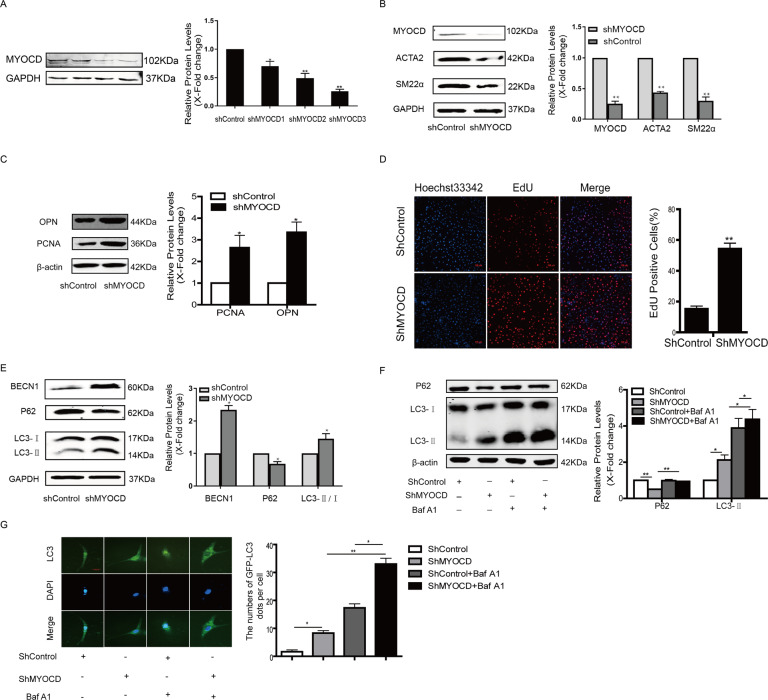
Knockdown of MYOCD triggered autophagy in HA-VSMCs. **A** The expression of MYOCD was detected in HA-VSMCs transfected with shRNA specifically targeting MYOCD by western blotting (*P < 0.05, **P < 0.01, *n* = 3). **B** The expression of contractile proteins ACTA2 and SM22 was detected in MYOCD-knockdown HA-VSMCs by western blotting (**P < 0.01, *n* = 3). **C** The expression of OPN and PCNA was tested in MYOCD-knockdown HA-VSMCs by western blotting (*P < 0.05, *n* = 3). **D** Cellular proliferation was evaluated in MYOCD-knockdown HA-VSMCs by EdU staining (**P < 0.01, *n* = 3). **E** The expression of beclin1, *P*62, and LC3 was tested in MYOCD-knockdown HA-VSMCs by western blotting (*P < 0.05, *n* = 3). **F** Bafilomycin A1 (0.1 μg/mL) was added to control or MYOCD-knockdown HA-VSMCs 1 h prior to harvest, and accumulation of P62 and LC3 was measured by western blotting (*P < 0.05,**P < 0.01, *n* = 3). **G** Representative images and quantification of GFP-LC3 puncta in MYOCD-knockdown HA-VSMCs transfected with GFP-LC3 vector in the presence or absence of bafilomycin A1 (*P < 0.05,**P < 0.01, *n* = 3).

To test whether MYOCD can regulate the autophagy response, MYOCD was knocked down in HA-VSMCs by transfection with an shRNA MYOCD plasmid. Knockdown of MYOCD was confirmed by western blotting (Fig. 1A), and a shRNA#3 plasmid against MYOCD was used in subsequent experiments. The expression of contractile proteins ACTA2 and SM22α was decreased in HA-VSMCs transfected with shRNA specifically targeting MYOCD compared with control shRNA (Fig. 1B), whereas the levels of proliferational markers Osteopontin (OPN) and proliferating cell nuclear antigen (PCNA) were increased (Fig. 1C), which was consistent with previous reports [3]. Knockdown of MYOCD significantly increased the number of EdU-positive cells (Fig. 1D). Knockdown of MYOCD decreased the expression of p62 and increased the expression of beclin1 and the conversion of LC3-I to LC3-II (Fig. 1E). Cotreatment of the lysosomal inhibitor bafilomycin A1 and shMYOCD led to higher accumulation of LC3-II and p62 compared to shMYOCD group (Fig. 1F), suggesting that knockdown of MYOCD increased the autophagic flux. Moreover, the formation of autophagosomes was observed in HA-VSMCs by transfecting cells with a green fluorescent protein (GFP)-LC3 expression construct and quantifying the number of GFP-LC3 puncta per cell by fluorescence microscopy. More GFP-LC3 dots were found in HA-VSMCs transfected with shRNA against MYOCD compared to HA-VSMCs transfected with control shRNA, and treatment of bafilomycin A1 in MYOCD-knockdown cells further increased the number of GFP-LC3 dots (Fig. 1G).

### Inhibition of autophagy in MYOCD-knockdown cells restored the expression of contractile proteins

**Fig. 2:**
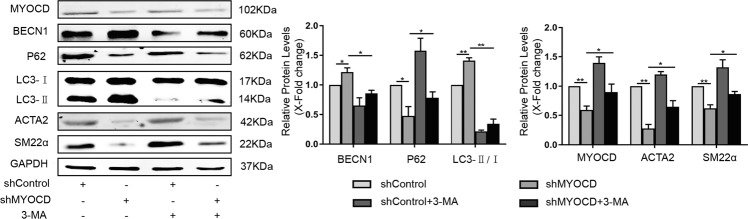
Inhibition of autophagy in MYOCD-knockdown cells restored the expression of contractile proteins. HA-VSMCs were transfected with shMYOCD plasmid for 4 h and then treated with 5 mM 3-MA for another 48 h. Western blot images and quantification in each group were shown (*P < 0.05, **P < 0.01, *n* = 3).

Next, we wanted to determine whether the expression of contractile proteins could be restored by 3-methyladenine (3-MA, an autophagy inhibitor). HA-VSMCs were transfected with sh-MYOCD plasmid for 4 h and then treated with 3-MA for another 48 h. As shown in Fig. 2, knockdown of MYOCD upregulated the expression of beclin1 and the ratio of microtubule-associated protein 1 light chain 3 alpha (LC3II/LC3I) and downregulated the level of p62, suggesting the activation of autophagy. The expression of the contractile proteins ACTA2 and SM22α was decreased in MYOCD knockdown cells. Treatment with 3-MA inhibited the autophagy induced by knockdown of MYOCD and restored the level of contractile proteins. These results showed that MYOCD could affect the expression of contractile proteins by regulating autophagy.

### MYOCD activated the transcription of miR-30a by binding to the CArG box

**Fig. 3:**
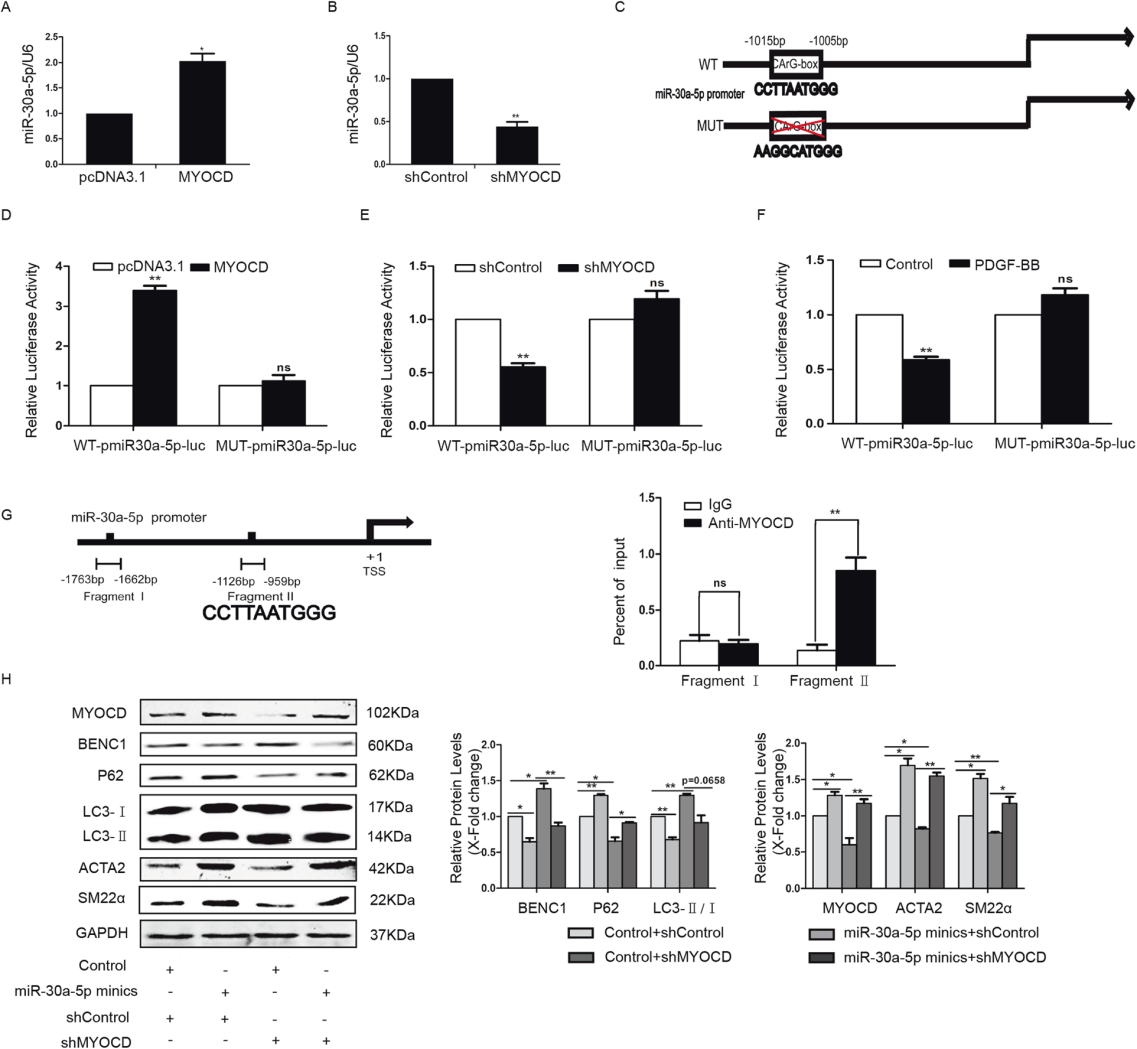
MYOCD activated the transcription of miR-30a by binding to the CArG box. The level of miR-30a-5p was examined in MYOCD overexpression (**A**) or knockdown (**B**) cells by RT-PCR (*P < 0.05, **P < 0.01, *n* = 3). **C** A schematic diagram of the miR-30a-5p promoter region represents wild-type and mutant CArG boxes. **D** Luciferase reporter plasmids together with pcDNA3.1-MYOCD plasmids or vectors were cotransfected into HA-VSMCs, and 24 h after transfection, luciferase activity was assayed (**P < 0.01, *n* = 3). **E** HA-VSMCs were transfected with luciferase reporter plasmids together with shControl or shMYOCD plasmids for 24 h, and then luciferase activity was assayed (**P < 0.01, *n* = 3). **F** HA-VSMCs were transfected with luciferase reporter plasmids in the presence or absence of PDGF-BB for 24 h, and then luciferase activity was assayed (**P < 0.01, *n* = 3). **G** 48 h after transfection of pcDNA3.1-MYOCD-myc vector expression plasmids or vector plasmids, ChIP assays were performed using antibodies against myc (**P < 0.01, *n* = 3). **H** MiR-30a-5p mimics were transfected into MYOCD knockdown cells, and then western blotting was performed (*P < 0.05, **P < 0.01, *n* = 3).

MiRNA-30a-5p has been known to mediate beclin1 suppression by targeting the 3′UTR of beclin1 [16]. Here, we presumed that MYOCD could positively regulate the expression of miR-30a-5p. To demonstrate this hypothesis, the level of miR-30a-5p was examined in MYOCD overexpression or knockdown cells by RT-PCR. As shown in Fig. 3A and B, overexpression of MYOCD led to an increase in miR-30a-5p, and knockdown of MYOCD led to a decrease in miR-30a-5p.

Bioinformatics analysis revealed that the promoter regions of miR-30a-5p contain a CArG box motif (CCTTAATGGG) (Fig. 3C). To confirm whether MYOCD can activate the transcription of miR-30a-5p, a luciferase reporter vector containing the promoter regions of miR-30a-5p was constructed, and then the luciferase assay was performed in MYOCD overexpression or knockdown cells. As shown in Fig. 3D, MYOCD activated the transcription of the miR-30a-5p promoter, while mutation of the CArG box in the miR-30a-5p promoter abolished this activation. The activity of the miR-30a-5p promoter was weakened by MYOCD knockdown or PDGF-BB treatment (Fig. 3E and F). To further confirm the specific binding of MYOCD to the miR-30a-5p promoter, ChIP assays were performed in cells transfected with the MYOCD-myc vector using antibodies against myc. As shown in Fig. 3G, MYOCD enrichment at the CArG box region of the miR-30a-5p promoter was significantly enhanced. These data demonstrated that MYOCD could promote the transactivation of miR-30a-5p by binding to the CArG box motif of the miR-30a-5p promoter.

Since knockdown of MYOCD can inhibit the level of miR-30a-5p, we wanted to know whether recovery of miR-30a-5p expression in MYOCD-knockdown cells could restore the expression of contractile proteins. As shown in Fig. 3H, miR-30a-5p mimics enhanced the expression of contractile proteins ACTA2 and SM22α and inhibited autophagy in MYOCD-knockdown cells.

### MiR-30a-5p decreased the expression of beclin1 by targeting the 3’UTR of beclin1

**Fig. 4:**
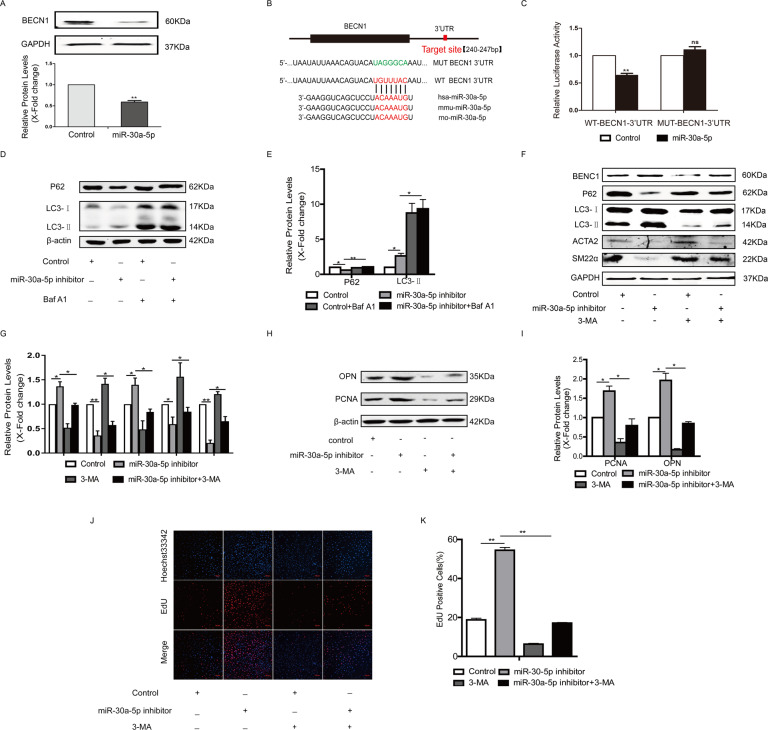
MiR-30a-5p decreased the expression of beclin1 by targeting the 3’UTR of beclin1. **A** HA-VSMCs were transfected with miR-30a-5p mimics, and then the expression of beclin1 was detected by western blotting (**P < 0.01, *n* = 3). **B** Alignment of the miR-30a-5p “seed region” with beclin1 3′-UTRs among different species. **C** HA-VSMCs were transfected with beclin1 3′-UTR or mutant beclin1 3′-UTR plasmids together with miR-30a-5p mimic or control for 24 h, followed by luciferase assays (**P < 0.01, *n* = 3). **D** and **E** HA-VSMCs were transfected with miR-30a-5p inhibitor or control for 24 h and then treated with bafilomycin A1 for 2 h. Cells were harvested and accumulation of P62 and LC3 was measured by western blotting (*P < 0.05, **P < 0.01, *n* = 3). **F**–**I** HA-VSMCs were transfected with miR-30a-5p inhibitor for 4 h and then treated with 5 mM 3-MA for another 48 h. Western blot images and quantification in each group are shown (*P < 0.05, **P < 0.01, *n* = 3). **J** and **K** Cellular proliferation was evaluated by EdU staining (**P < 0.01, *n* = 3).

To confirm that beclin1 is a target of miR-30a-5p in HA-VSMCs, cells were transfected with miR-30a-5p mimics, and then the expression of beclin1 was detected by western blotting. The results showed that miR-30a-5p significantly reduced the expression of beclin1 in HA-VSMCs (Fig. 4A). To confirm that miR-30a-5p can target the 3′-untranslated region (3′-UTR) of beclin1 mRNA to suppress protein translation, the 3′-UTR of beclin1 containing miR-30a-5p binding sites (WT-BECN1-3’UTR) or mutation (MUT-BECN1-3′UTR) was cloned into the pmirGLO vector (Fig. 4B), and this vector was cotransfected with the miR-30a-5p mimics in cells for 24 h. As indicated in Fig. 4C, the overexpression of a miR-30a-5p mimic repressed the activity of luciferase fused with the WT-3′-UTR of beclin1 but had no effect on luciferase activity when the miR-30a-5p binding sites in the 3′-UTR of beclin1 were mutated. Consistent with other reports [14–16], our results also confirmed that miR-30a-5p could negatively regulate the level of beclin1 in HA-VSMCs by targeting the 3′-UTR of beclin1.

Then, we hypothesized that miR-30a-5p knockdown could activate autophagy and cause a decrease in the level of contractile proteins. As shown in Fig. 4D and E, the autophagic flux was measured in miR-30a-5p inhibitor-treated cells with/without bafilomycin A1 by detecting the levels of p62 and LC3. The results showed that cotreatment of bafilomycin A1 and miR-30a-5p inhibitor led to higher accumulation of LC3-II and p62 compared to miR-30a-5p inhibitor group, suggesting that miR-30a-5p inhibitor activates autophagy. Furthermore, decreased expression of the contractile proteins ACTA2 and SM22α was observed in cells transfected with the miR-30a-5p inhibitor (Fig. 4F and G). Incubation with 3-MA inactivated autophagy and enhanced the expression of contractile proteins in cells transfected with the miR-30a-5p inhibitor (Fig. 4F and G). MiR-30a-5p inhibitor also increased the expression of PCNA and OPN, while 3-MA treatment inhibited the expression of PCNA and OPN (Fig. 4H and I). More EDU positive cells were observed in miR-30a-5p inhibitor group, compared with Control group, while 3-MA treatment reversed this change caused by miR-30a-5p inhibitor (Fig. 4J and K).

### Inhibition of autophagy could maintain the contractile phenotype in PDGF-BB-stimulated HA-VSMCs

**Fig. 5:**
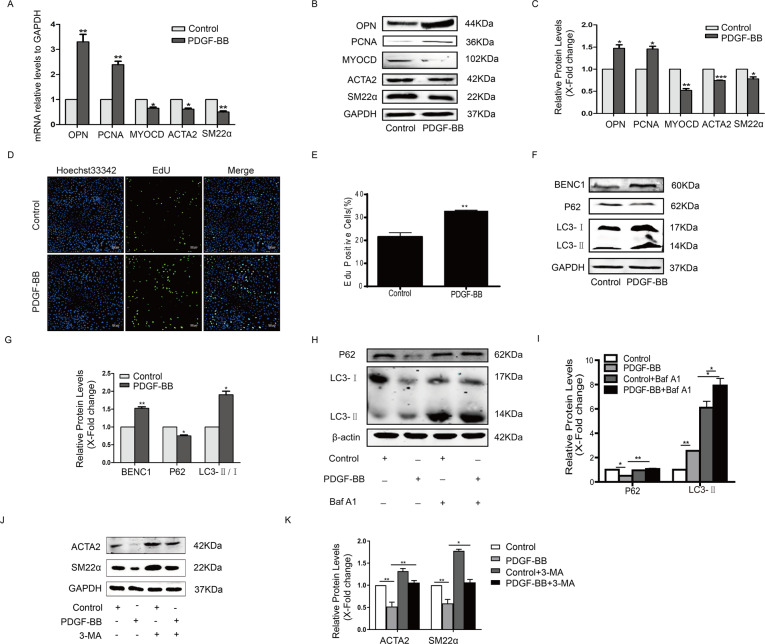
Inhibition of autophagy maintained the contractile phenotype in PDGF-BB-stimulated HA-VSMCs. **A** HA-VSMCs were starved overnight prior to stimulation using 40 ng/mL PDGF-BB for 24 h, and then the mRNA levels of OPN, PCNA, MYOCD, SM22α and ACTA2 were detected by qRT-PCR (*P < 0.05, **P < 0.01, *n* = 3). **B** and **C** The protein levels of these proliferative markers and contractile genes were detected in HA-VSMCs treated with 40 ng/mL PDGF-BB for 48 h by western blotting (*P < 0.05, **P < 0.01, ***P < 0.001, *n* = 3). **D** and **E** Cellular proliferation was evaluated in PDGF-BB-stimulated HA-VSMCs by EdU staining (**P < 0.01, *n* = 3). **F** and **G** The expression of beclin1, P62 and LC3 was detected in PDGF-BB-stimulated HA-VSMCs by western blotting (*P < 0.05, **P < 0.01, *n* = 3). **H** and **I** HA-VSMCs were incubated with PDGF-BB for 24 h and then treated with bafilomycin A1 for 2 h. Cells were harvested and accumulation of P62 and LC3 was measured by western blotting (*P < 0.05,*P < 0.01, *n* = 3). **J** and **K** Serum-starved HA-VSMCs were pretreated with 5 mM 3-MA for half an hour and then incubated with 40 ng/mL PDGF-BB for another 48 h. Western blot images and quantification in each group are shown (*P < 0.05, **P < 0.01, *n* = 3).

PDGF-BB plays a major role in inducing the switch of VSMCs from the contractile phenotype to the proliferative phenotype. Here, PDGF-BB stimulation upregulated the expression of the proliferative markers OPN and PCNA, and downregulated the expression of ACTA2 and SM22α in HA-VSMCs (Fig. 5 A–C). The PDGF-BB-stimulated switch of HA-VSMCs into a proliferative phenotype was also confirmed by EdU staining (Fig. 5D and E). In line with previous studies [10], we also found that PDGF-BB triggered autophagy in HA-VSMCs, as confirmed by the increase in beclin1 protein and LC3II/LC3I and the decrease in P62 protein (Fig. 5F and G). PDGF-BB induced a significant increase in LC3-II and p62 expression, when bafilomycin A1 blocked lysosomal degradation (Fig. 5H and I). Inhibition of autophagy with 3-MA stabilized the expression of SM22α and ACTA2 in PDGF-BB-treated HA-VSMCs (Fig. 5J and K). These results indicated that PDGF-BB could induce HA-VSMC proliferation and trigger autophagy, while inhibition of autophagy could maintain the contractile phenotype.

### Restoring the expression of miR-30a-5p in PDGF-BB-treated cells upregulated the level of contraction proteins

**Fig. 6:**
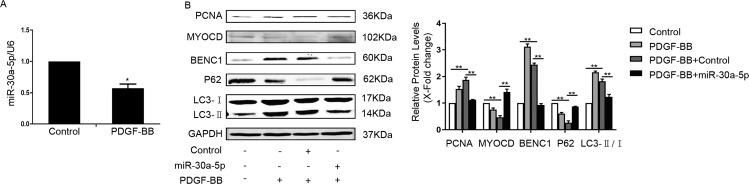
Restoring the expression of miR-30a-5p in PDGF-BB-treated cells upregulated the level of contraction proteins. **A** Serum-starved HA-VSMCs were treated with 40 ng/mL PDGF-BB for 24 h, and then the level of miR-30a-5p was detected by qRT-PCR (*P < 0.05, *n* = 3). **B** Serum-starved HA-VSMCs were transfected with miR-30a-5p mimics for 4 h and then stimulated with 40 ng/mL PDGF-BB for 48 h. Western blot images and quantification in each group are shown (*P < 0.05, **P < 0.01, *n* = 3).

We next investigated the expression of miR-30a-5p in cells stimulated by PDGF-BB. As shown in Fig. 6A, miR-30a-5p was significantly decreased in PDGF-BB-stimulated cells. Because miR-30a-5p could inactivate autophagy, we hypothesized that inhibition of autophagy induced by miR-30a-5p could restore the levels of SM22α and ACTA2 in PDGF-BB-stimulated cells. As shown in Fig. 6B, miR-30a-5p reversed the effect of PDGF-BB in HA-VSMCs. MiR-30a-5p increased the accumulation of p62 and repressed the expression of beclin1 and the ratio of LC3II/LC3I, indicating the inhibition of autophagy. The loss of contractile proteins due to PDGF-BB treatment was resisted by miR-30a-5p. These results showed that miR-30a-5p could maintain the contractile phenotype of HA-VSMCs by inhibiting excessive autophagy.

### Restoration of miR-30a-5p in the injured site abolished neointimal formation caused by wire injury of carotid arteries

**Fig. 7:**
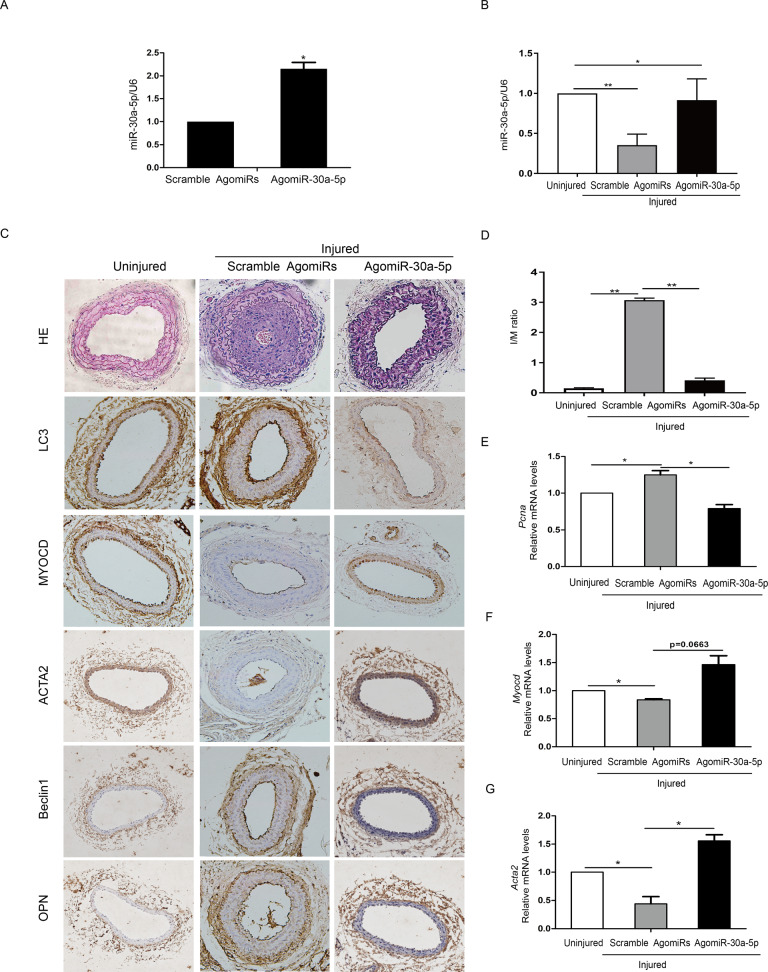
Restoration of miR-30a-5p in the injured site abolished neointimal formation caused by wire injury of carotid arteries. Wire injury was performed in vivo on mouse carotid arteries, and then local perivascular delivery of miR-30a-5p agomiRs or scramble agomiRs was carried out. **A** Three days after injury, injured segments of femoral arteries were harvested, and qRT-PCR was performed to confirm the overexpression of miR-30a-5p (*P < 0.05, n = 4–6). **B** At 21 days postinjury, injured segments of femoral arteries were harvested and subjected to qRT-PCR to detect the level of miR-30a-5p (*P < 0.05, **P < 0.01, *n* = 4–6). **C** Representative images of HE staining and histochemistry staining. **D** Delivery of miR-30a-5p agomiRs led to a reduction in neointima size and a reduction in the intimato-media ratio (**P < 0.01, *n* = 4–6). The mRNA levels of *Pcna* (**E**), *Myocd* (**F**) and *Acta2* (**G**) were detected in injured mouse carotid arteries with miR-30a-5p agomiRs or scramble agomiRs by qRT-PCR (*P < 0.05, *n* = 4–6).

To further confirm the role of miR-30a-5p in VSMC phenotype switching, we performed a wire injury procedure on mouse carotid arteries, followed by local perivascular delivery of miR-30a-5p agomiRs or scramble agomiRs. Three days or 21 days later, injured segments of femoral arteries were harvested and subjected to qRT-PCR and histochemistry staining. Three days after wire injury and local delivery of AgomiRs, the level of miR-30a-5p was detected in carotid arteries by qRT-PCR. Perivascular delivery of AgomiR-30a-5p significantly increased the levels of miR-30a-5p in the injured vessels (Fig. 7A). Then, we detected the expression of miR-30a-5p in the injured carotid arteries 21 days after wire injury. Importantly, the expression levels of miR-30a-5p were also significantly downregulated in the injured versus uninjured arteries (Fig. 7B). As shown in Fig. 7C, extensive neointimal growth was observed in sections of injured carotid arteries of mice treated with scramble AgomiRs compared with control mice. Treatment with agomiR-30a-5p effectively inhibited neointimal formation and the expression of LC3 and beclin-1 in the injured portion (Fig. 7C). AgomiR-30a-5p also increased the levels of MYOCD and ACTA2, and meanwhile decreased the levels of OPN. AgomiR-30a-5p decreased wire injury-induced neointimal hyperplasia, the intimato-media ratio and stenosis (Fig. 7D). In comparison with uninjured arteries, injured arteries treated with scramble AgomiRs displayed a significant increase in the level of the proliferation marker PCNA (Fig. 7E) and a decrease in the levels of the differentiation markers MYOCD and ACTA2 (Fig. 7F and G). Treatment with AgomiR-30a-5p restored the levels of MYOCD and ACTA2 and decreased smooth muscle cell proliferation, as confirmed by the decreased expression of PCNA (Fig. 7E–G). These results showed that restoration of miR-30a in the injured site inhibited the SMC phenotype switch and the neointimal formation caused by wire injury of carotid arteries.

## Discussion

By performing MYOCD loss-of-function studies, we demonstrated that MYOCD knockdown triggered autophagy in HA-VSMCs, as indicated by an increased LC3-II/LC3-I ratio, LC3 puncta formation and beclin1 expression but decreased p62 accumulation. This is consistent with the recent literature showing that SMC-restricted MYOCD mutant mice exhibit activation of autophagy and deformation of the arterial structure [4]. MYOCD deletion can also trigger autophagy in primary cultures of mouse aortic SMCs in vitro, as confirmed by robust induction of beclin-1, autophagy related 5 (ATG5), (autophagy related 7) ATG7, and LC3 [4]. Simultaneously, MYOCD knockdown also led to downregulation of SMC contractile genes and the loss of the contractile SMC phenotype. Thus, we hypothesized that autophagy induced by MYOCD knockdown led to the degradation of SMC contractile proteins. For VSMCs, autophagy-lysosome, instead of ubiquitin-proteasome, could be the major proteolytic pathway to remove contractile proteins [10].

PDGF-BB [10], TNF-α [9], nicotine [13], and shear stress [11] have been reported to promote VSMC phenotype switching and induce autophagy. These factor-induced phenotypic changes could depend on autophagy because inhibition of autophagy by treatment with 3-methyladenine (3-MA) reversed these effects and maintained the differentiation phenotype of VSMCs. Similarly, we found that MYOCD knockdown-mediated autophagy activation and VSMC phenotype switching were reversed by treatment with the autophagy inhibitor 3-MA. This result was coincident with several previous findings that activation of autophagy was observed in neointimal lesions of mouse common carotid arteries and that 3-MA prevented neointima formation [17]. Although our findings, together with other studies, have confirmed that deletion or knockdown of MYOCD could activate autophagy, the mechanism by which MYOCD regulates autophagy activities remains to be elucidated.

miR-30a-5p was found to suppress the expression of beclin1 in hepatic stellate cells [18], cardiomyocytes [19–21], and a variety of tumor cells [14–16], leading to the inhibition of autophagy. A recent study confirmed that miR-30a inhibits autophagy and promotes VSMC senescence in rat aortic VSMCs by regulating the expression of beclin1 [22]. Consistent with a previous study, we demonstrated that beclin1 is a target of miR-30a-5p in HA-VSMCs. Using WT-3′UTR-BECN1 and mutant-3′UTR-BECN1 luciferase reporter vectors, we found that miR-30a-5p could repress the level of beclin1 by targeting the 3′UTR of beclin1. However, it is not known whether the expression of miR-30a-5p is regulated by MYOCD, a powerful transcriptional coactivator of SRF. By performing MYOCD loss-of function or gain-of function studies, we found that the expression of MYOCD was positively correlated with the level of miR-30a-5p in HA-VSMCs. We specifically showed that miR-30a-5p is transcriptionally regulated by MYOCD. MYOCD could activate the transcription of miR-30a-5p by binding to the CArG box of its promoter, as confirmed by luciferase reporter assays and ChIP. Thus, MYOCD could inactivate autophagy by activating miR-30a-5p and thus inhibiting beclin1.

To further clarify whether the effect of MYOCD in regulating autophagy depends on miR-30a-5p, miR-30a-5p mimics were transfected into MYOCD-knockdown cells. The data showed that miR-30a-5p mimics effectively restrained excessive autophagy induced by MYOCD knockdown and restored the expression of contractile proteins. It has been reported that miR-30a-5p could inhibit the activation of nuclear factor κB (NF-κB) in many types of cells [23–25]. NF-κB activation has also been shown to inhibit myocardin expression and myocardin-mediated smooth muscle contractile marker genes [26–29]. Thus, miR-30a-5p could increase the levels of myocardin by inhibiting the activation of NF-κB. Furthermore, the miR-30a-5p inhibitor activated autophagy in HA-VSMCs, and 3-MA treatment inhibited activated autophagy and resumed the expression of contractile proteins.

PDGF-BB is a potent mitogen for VSMCs, acting on the PDGF-beta receptor in VSMCs and promoting the migration and proliferation of VSMCs. MYOCD is inhibited by PDGF-BB signaling via transcription factors such as Kruppel-like factor 4 [30, 31] and ETS-like gene 1 (Elk-1) [31, 32] or miRNAs [33]. Several recent studies have suggested that autophagy is required for PDGF-BB-induced phenotypic conversion of SMCs [34–36]. PDGF-BB initiated autophagy in VSMCs through a 5′ adenosine monophosphate-activated protein kinase-independent and mammalian target of rapamycin-independent mechanism, leading to the rapid degradation of contractile proteins [10]. All three inhibitors of autophagy, i.e., spautin-1,3-MA and bafilomycin, prevented PDGF-BB-induced losses of contractile proteins and maintained the contractile phenotype [10]. In line with this study, our data showed that treatment with PDGF-BB enhanced the expression of the proliferative markers OPN and PCNA and reduced the levels of MYOCD, SM22α, and ACTA2 in HA-VSMCs. PDGF-BB induced cellular proliferation, as confirmed by EdU staining. This was accompanied by the activation of autophagy, characterized by an increase in beclin1 and LC3II/LC3I and a decrease in p62. These effects of PDGF-BB were partially reversed by 3-MA.

Accumulating evidence show that autophagy activation in VSMCs is an essential regulator of the transition from a contractile to a synthetic phenotype, which contributes to neointima formation. High autophagy is observed not only in PDGF-stimulated VSMCs in vitro but also in the VSMCs of injured arteries in vivo using a mouse partial left carotid artery (PLCA) ligation-injury model [37]. Treatment with 3-MA significantly alleviates PLCA ligation-induced neointima formation and decreased the cell proliferation marker PCNA in mice [37]. Furthermore, autophagy is overactivated in carotid arteries after balloon injury and that inhibition of autophagy activation contributes to preventing VSMC phenotype switching and hyperproliferation [37, 38]. Jumonji domain-containing protein 3 (JMJD3) and mesoderm/mesenchyme homeobox gene l (Meox1) were found to be novel inducers of SMC phenotypic switching. Knockdown of JMJD3 or Meox1 attenuated injury-induced neointima formation and inhibited VSMC hyperproliferation in a rat carotid artery balloon injury model by suppressing autophagic activation.

However, basal autophagy levels are critically required for VSMC survival and vascular integrity during the development and progression of AAs. A recent study using TaglnCre^+^/Atg5^flox/flox^ mice suggested that loss of basal autophagy in VSMCs increased the susceptibility of VSMCs to death, enhanced ER stress activation, and promoted VSMC inflammation [7]. They also observed the induction of autophagy and endoplasmic reticulum stress in VSMCs of human dissecting AAs. Similarly, autophagic activity was increased in the aortic wall of thoracic aortic dissection patients [36]. Therefore, abnormal autophagy could regulate the functional properties of aortic SMCs, which might be associated with the potential pathogenesis of intimal hyperplasia-related vascular diseases.

In the present study, we found that miR-30a-5p was significantly downregulated in PDGF-BB-treated HA-VSMCs in vitro and wire injury-induced mouse carotid arteries in vivo. Liu et al. observed that the expression of miR-30 family members, including miR-30a-e, was significantly reduced in balloon-injured rat carotid arteries compared with uninjured rat carotid arteries [39]. Lentiviral delivery of miR-30c into injured carotid arteries prevented SMC proliferation and neointima formation by inhibiting the activation of CaMKIIδ2 [39]. Another study suggested that downregulation of miR-29b induced VSMC phenotypic modulation by directly activating ATG14-mediated autophagy, which was involved in the pathogenesis of intracranial aneurysm. Importantly, we found that restoring the expression of miR-30a-5p in PDGF-BB-treated HA-VSMCs could stabilize the expression of contractile proteins by inhibiting excessive autophagy. With a wire-injured mouse carotid artery model, we confirmed that local perivascular delivery of miR-30a-5p AgomiRs inhibited neointima formation by inhibiting the activation of autophagy.

In summary, we showed that MYOCD regulated the expression of miR-30a-5p, a potent inhibitor of autophagy, by downregulating beclin1 expression. Our study provided the first evidence that MYOCD knockdown induced the degradation of VSMC contractile proteins by suppressing miR-30a-5p and activating autophagy. Restoring the expression of miR-30a-5p prevented PDGF-BB-induced VSMC phenotype modulation in vitro and inhibited VSMC proliferation in the arterial walls and neointimal hyperplasia in vivo following carotid injury. These studies provide a new potential therapeutic strategy to reduce the progression of intima hyperplasia-related vascular diseases.

## Materials and methods

### Cell culture and treatment

Human aortic VSMCs (HA-VSMCs) were obtained from the American Type Culture Collection (ATCC, Manassas, VA) and cultured in Dulbecco’s modified high glucose medium (DMEM-HG) (Gibco, New York, USA) containing 10% fetal bovine serum (AusGeneX, Gold Coast, Australia) at 37 °C in 5% CO_2_ and 95% air.

HA-VSMCs were seeded onto 6-well plates at 1 × 10^6^ cells/mL. The cells were starved in serum-free media for another 24 h and then subsequently treated with 40 ng/mL PDGF-BB (R&D, Minneapolis, USA) for 48 h.

### Plasmid DNA and miRNA transfection

A MYOCD-targeting shRNA plasmid (shMYOCD), a scrambled control shRNA plasmid (shControl), a pcDNA3.1-MYOCD expression plasmid (MYOCD) and an empty vector pcDNA3.1 (pcDNA3.1) have been described previously [40, 41]. The GFP-LC3 plasmid was a gift from Congcong He (Northwestern University, Chicago, USA). Plasmid DNA transfection was carried out using X-tremeGENE HP DNA transfection reagent (Roche, Basel, Switzerland) according to the manufacturer’s instructions. MiR-30a-5p mimics, inhibitors and miRNA negative controls were purchased from RiboBio (RiboBio, Guangzhou, China). MiRNA transfection was performed using riboFect™ CP reagent according to the manufacturer’s instructions.

### Western blotting

Whole-cell proteins were extracted and subjected to standard western blot analysis as described previously [41]. Antibodies against MYOCD (SAB4200539, Sigma, Massachusetts, USA), ACTA2(ab7817, Abcam, Massachusetts, USA), SM22α (ab14106, Abcam, Massachusetts, USA), PCNA (sc-56, Santa Cruz, California, United States), OPN (sc21742, Santa Cruz, California, United States), LC3 (NB100-2220, Novus Biologicals, Colorado, USA), P62 (610833,BD Transduction Laboratories, USA), Beclin1 (ab207612, Abcam, Massachusetts, USA) and glyceraldehyde-3-phosphate dehydrogenase (GAPDH) (ab8245, Abcam, Massachusetts, USA) were used to probe for the target proteins. Secondary antibodies, including IRDye^®^800CW anti-rabbit secondary antibody and IRDye^®^680 anti-mouse secondary antibody, were purchased from Li-COR (Lincoln, Michigan, USA). Signals were detected by an Odyssey™ Infrared Imaging System (LI-COR, Michigan, USA), and the bands were quantified by Image-Pro Plus 5.1 software (MEDIA CYBERNETICS, Maryland, USA).

### Quantitative real-time PCR (qRT-PCR)

Real-time PCR for mRNA and miRNA was performed as described in our previous studies in an Applied Biosystems real-time PCR system [41]. Briefly, total RNA was extracted using TRIzol reagent (Invitrogen, California, USA), and small RNA was extracted using a miRcute miRNA isolation kit (TIANGEN, Beijing, China) according to the manufacturer’s instructions. cDNA was reverse transcribed from total RNA using M-MLV reverse transcriptase (Promega, Minnesota, USA) with random primers or specific primers for miR-30a-5p and U6. The sequences of reverse transcription primers were as follows: miR-30a-5p: GTCGTATCCAGTGCAGGGTCCGAGGTATTCGCACTGGATACGACCTTCCAG; U6:GTCGTATCCAGTGCAGGGTCCGAGGTATTCGCACTGGATACGACAAAAT ATG. Bestar® SybrGreen qPCR mastermix (DBI Bioscience, Ludwigshafen, Germany) was used for single-step qRT-PCRs. The results were standardized to control values of GAPDH or U6. The PCR primer sequences are listed in Table 1.

**Table 1:** The PCR primer sequences.

Gene name	Sequence (5′→3′)
***GAPDH***	F-ATTCAACGGCACAGTCAAGG
	R-GCAGAAGGGGCGGAGATGA
U6	F-CTCGCTTCGGCAGCACA
	R-AACGCTTCACGAATTTGCGT
Myocardin	F-AGTAAGAACCGCCACAAA
	R-GAGCATAGGCAGAGTCCA
PCNA	F-GGCTCCATCCTCAAGAAGGTGTT
	R-CGTTATCTTCGGCCCTTAGTGTA
ACTA2	F-AGCCAAGCACTGTCAGGAATC
	R-GAGCCCAGAGCCATTGTCAC
OPN	F-AGTACCCTGATGCTACAGACGAG
	R-CGTTTCATAACTGTCCTTCCCAC
miR-30a-5p	F-GAGAGCTGTAAACATCCTCGA
	R-GCAGGGTCCGAGGTATTC
miR-30a-5p reverse transcription primer	GTCGTATCCAGTGCAGGGTCCGAGGTATTCGCACTGGATACGACCTTCCAG

### Immunofluorescence assay

For quantification of GFP-LC3 dots, HA-VSMCs were cotransfected with shMYOCD or shControl together with GFP-LC3 plasmid for 48 h. The numbers of GFP-LC3 dots per cell were quantified from three independent experiments (≥100 cells per experiment).

### Luciferase reporter assay

For gene promoter activity assays, the promoter regions of miR-30a-5p were amplified by PCR and then cloned into the pGL3 luciferase reporter vector (WT-pmiR30a-5p-luc). The primers used were as follows: 5′-AGAGCTAGCGAGTAGTATAGGTCCCACTTGGAT-3′ (sense) and 5′-CTTCTCGAGATACCTTCTTTAGCCTTCTGTTGGG-3′ (antisense). Luciferase reporter constructs containing the CArG box-mutated hsa-miR-30a-5p promoter (MUT-pmiR30a-5p-luc) were generated using a QuikChange site-directed mutagenesis kit (Stratagene, La Jolla, CA). The primers used were as follows: 5′-GACTCAAAGATTAACTTTTTTAAAAGGCATGGGATAGAGTTACCCTTTGCTTT-3′ (sense); and 5′-AAAGCAAAGGGTAACTCTATCCCATGCCTTTTAAAAAAGTTAATCTTTGAGTC-3′ (antisense). The vector pGL3 was used as a control. VSMCs were cotransfected with gene promoter plasmids (WT-pmiR30a-5p-luc or MUT-pmiR30a-5p-luc) and the expression plasmids as indicated in the figure legends using X-tremeGENE HP DNA transfection reagent (Roche, Basel, Switzerland). Luciferase assays were performed using a luciferase assay kit (Promega, Minnesota, USA) according to the manufacturer’s protocol. For beclin1 3′UTR reporter activity assays, luciferase-UTR reporter constructs (WT-BECN1-3′UTR) were generated by inserting the beclin1 3′-UTR. The primers used for PCR were as follows: 5′-GTTTAAACGAGCTCGCTAGCCTTTTTTCCTTAGGGGGAGGTTTG-3′ (sense) and 5′-CTCTAGACTCGAGGCTAGCGGCAGTTTTCAGACTGCAGCAAAT-3′ (antisense). The beclin1 3′UTR-MUT luciferase reporter plasmid (MUT-BECN1-3′UTR) was constructed using the following primers: 5′-ATTCGGGTAATATTAAACAGTACATAGGGCAAATACCAAAAAAGAAAAAATC-3′ (sense) and 5′-GATTTTTTCTTTTTTGGTATTTGCCCTATGTACTGTTTAATATTACCCGAAT-3′ (antisense). HA-VSMCs were cotransfected with reporter plasmids (WT-BECN1-3′UTR or MUT-BECN1-3′UTR) and control or miR-30a-5p mimics using X-tremeGENE HP DNA transfection reagent. After 24 h, luciferase activity was measured with a Synergy™ 4 (Bioteck, Vermont, USA). Transfection efficiencies were normalized by the total protein concentrations of each sample.

### Chromatin immunoprecipitation (ChIP) assays

ChIP analysis was performed as described previously [41]. The crosslinking reaction was terminated by glycine in MYOCD-transfected cells treated with 1% formaldehyde. The DNA fragments were broken into 200–1000 bp fragments by ultrasonic disruption. Protein–DNA complexes were immunoprecipitated using primary antibodies against myc (MYOCD-myc) (Proteintech, Chicago, USA). Samples were immunoprecipitated with nonimmune rabbit IgG (Beyotime Biotechnology, Shanghai, China) as a negative control. After washing and reversing the cross-links, the enriched DNA was purified and then quantified by real-time PCR. Primer sequences for amplification of the miR-30a-5p promoter containing one CArG box were as follows: 5′-ATAAAGAAAAAGGCCACA-3′ (sense) and 5′- AAAATCCACACAAAAAGC-3′ (antisense).

### EdU staining

Cell proliferation was determined by the incorporation of 5-ethynyl-20-deoxyuridine (EdU) with a Cell-Light^TM^ EdU DNA Cell Proliferation Kit (RiboBio, Guangzhou, China). Briefly, after being treated with 40 ng/mL PDGF-BB for 48 h, the cells were incubated in 100 μL of 50 μM EdU for 2 h, fixed with 4% paraformaldehyde, and stained with Hoechst 33342. The cells were observed under a laser confocal microscope (OLYMPUS, Tokyo, Japan), and the rate of EdU-positive cells/total cells was counted.

### A mouse model of wire-injured carotid artery and perivascular delivery of miR-30a-5p agomiR

Male C57BL/6J (8 weeks old) mice were purchased from the PLA Military Academy of Medical Sciences Laboratory Animal Center (Beijing, China) and were randomly divided into three groups. All animal procedures were performed in accordance with the National Institutes of Health Guide for the Care and Use of Laboratory Animals (the 8th Edition, revised in 2011) and approved by the Animal Ethics Committee of Tianjin University of Science and Technology. Wire-induced carotid artery injury was performed as described previously [3]. Briefly, mice were anesthetized by pentobarbital (70 mg/kg) via intraperitoneal injection. The left carotid artery of the anesthetized mouse was exposed and looped with silk sutures to temporarily stop the blood flow. A small incision was made, and a metal guide wire (0.381 mm in diameter) (Cook Inc., Bloomington, USA) was inserted into the artery and moved in and out 5 times with rotation to denude the endothelium. After vascular injury, local delivery of AgomiRs was performed according to previous reports [37]. Briefly, 100 μL of 30% pluronic F-127 gel (Beyotime, Guangzhou, China) containing 2.5 nmol miR-30a-5p or scramble AgomiRs was applied perivascularly to the injured carotid arteries for local delivery of AgomiRs. Twenty-one days after the injury, the mice were euthanized, and their carotid arteries were isolated and fixed with 4% paraformaldehyde or frozen directly in liquid nitrogen. The tissues were stained with hematoxylin and eosin for analysis of neointimal growth or stained with anti-LC3 antibody for evaluation of autophagy. The intima/media ratio of randomly selected sections of the carotid arteries was analyzed. qRT-PCR was performed to detect the mRNA levels of PCNA, MYOCD, and ACTA2.

### Statistical analysis

All of the data were presented as the means ± S.E.M. The differences between two experimental groups were analyzed by using a two-tailed Student’s *t* test or one-way ANOVA. *P* < 0.05 was considered statistically significant and expressed as *P < 0.05, **P < 0.01, ***P < 0.001.

## Supplementary information

Original data of western blot for Figure 1

Original data of western blot for Figure 2

Original data of western blot for Figure 3

Original data of western blot for Figure 4

Original data of western blot for Figure 5

Original data of western blot for Figure 6

Reproducibility checklist

## Data Availability

The data used to support the findings of this study are available from the corresponding author upon request.
