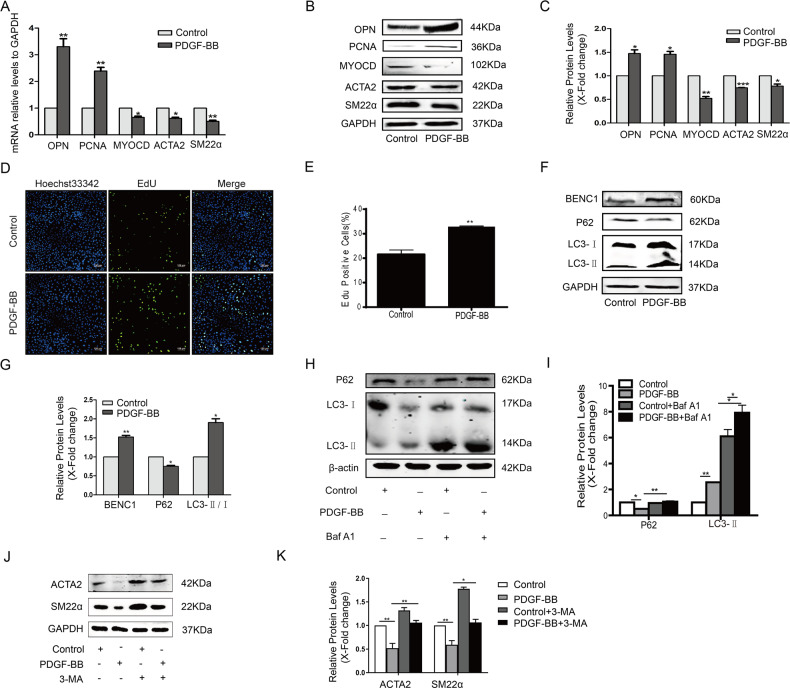# Correction: Myocardin/microRNA-30a/Beclin1 signaling controls the phenotypic modulation of vascular smooth muscle cells by regulating autophagy

**DOI:** 10.1038/s41419-022-04849-y

**Published:** 2022-04-19

**Authors:** Danyang Shi, Jinhua Ding, Shouqiang Xie, Lei Huang, Hongmin Zhang, Xiaojie Chen, Xuejun Ren, Sa Zhou, Hongpeng He, Wenjian Ma, Tongcun Zhang, Nan Wang

**Affiliations:** 1 College of Biotechnology, Tianjin University of Science and Technology, Tianjin, China; 2 Key Laboratory of Industrial Fermentation Microbiology, Ministry of Education and Tianjin, Tianjin, China; 3 Department of Cardiology, Beijing Anzhen Hospital Affiliated to Capital Medical University, Beijing, China

**Keywords:** Autophagy, Cell growth, miRNAs

Correction to: *Cell Death & Disease* 10.1038/s41419-022-04588-0, published online 08 February 2022

The original version of this article unfortunately contained two errors. For figure 5D an incorrect image was used during the figure preparation. The corrected figure can be found below. In addition, the product number of the antibody to myocardin (MYOCD) should be SAB4200539. The authors apologize for the errors. The original article has been corrected.