# Supplementary figures and images for: Myocardin/microRNA-30a/Beclin1 signaling controls the phenotypic modulation of vascular smooth muscle cells by regulating autophagy
Source: Cell Death Dis. 2022 Feb 8;13(2):121. doi: 10.1038/s41419-022-04588-0 (PMC8827084; doi:10.1038/s41419-022-04588-0)

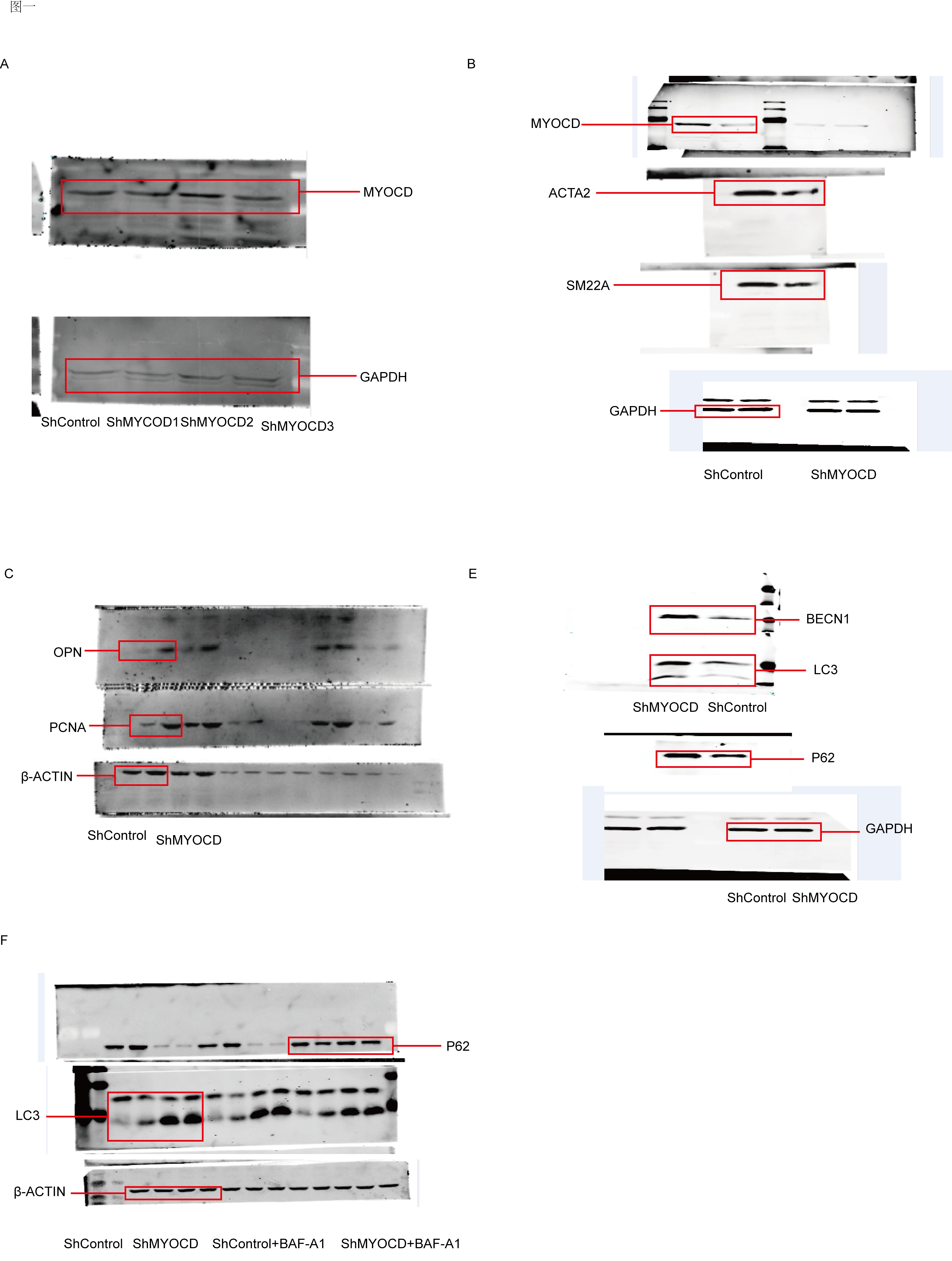

Supplement: Supplementary file 1 — Original data of western blot for Figure 1 [file 41419_2022_4588_MOESM1_ESM.png]

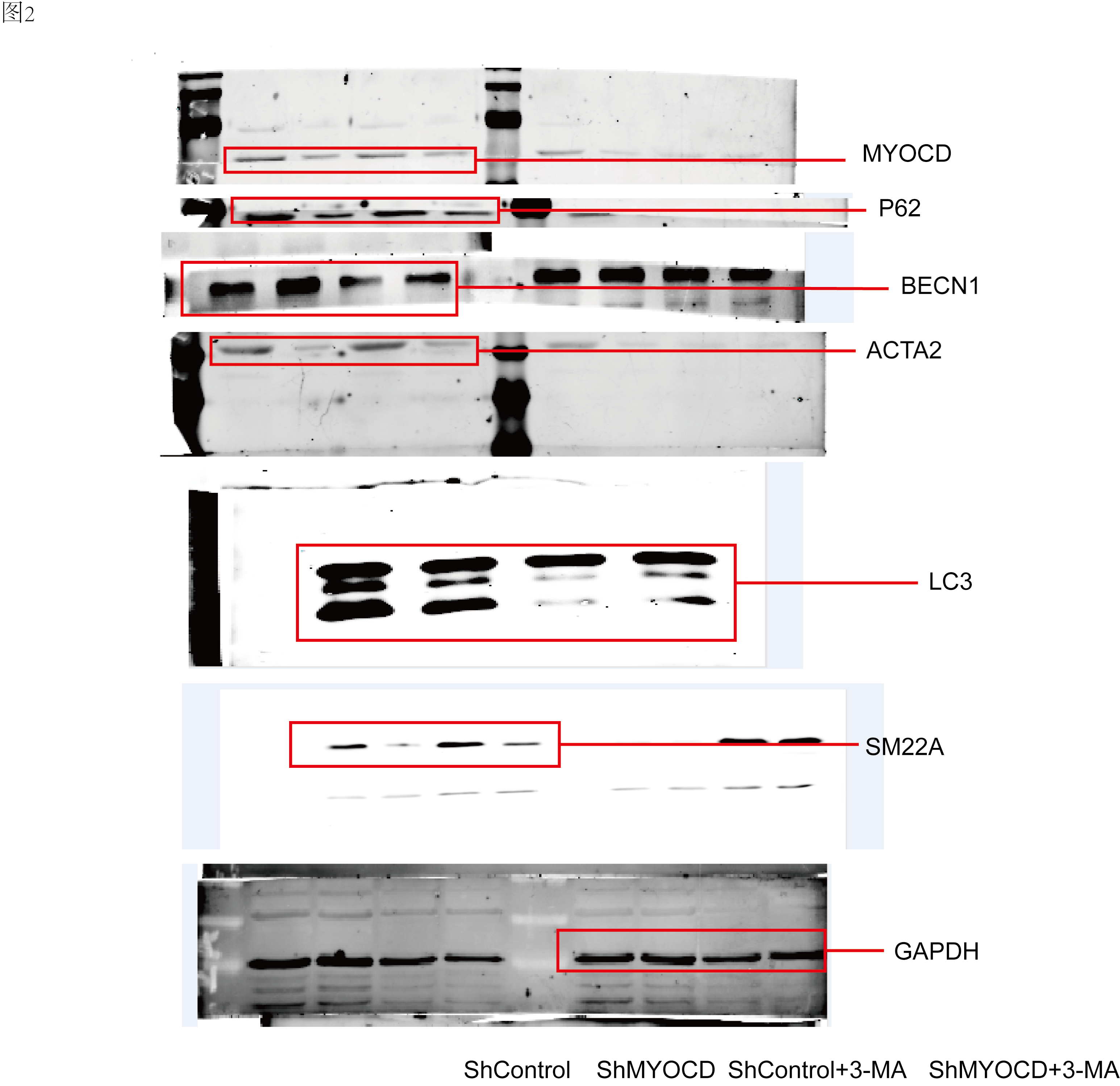

Supplement: Supplementary file 2 — Original data of western blot for Figure 2 [file 41419_2022_4588_MOESM2_ESM.png]

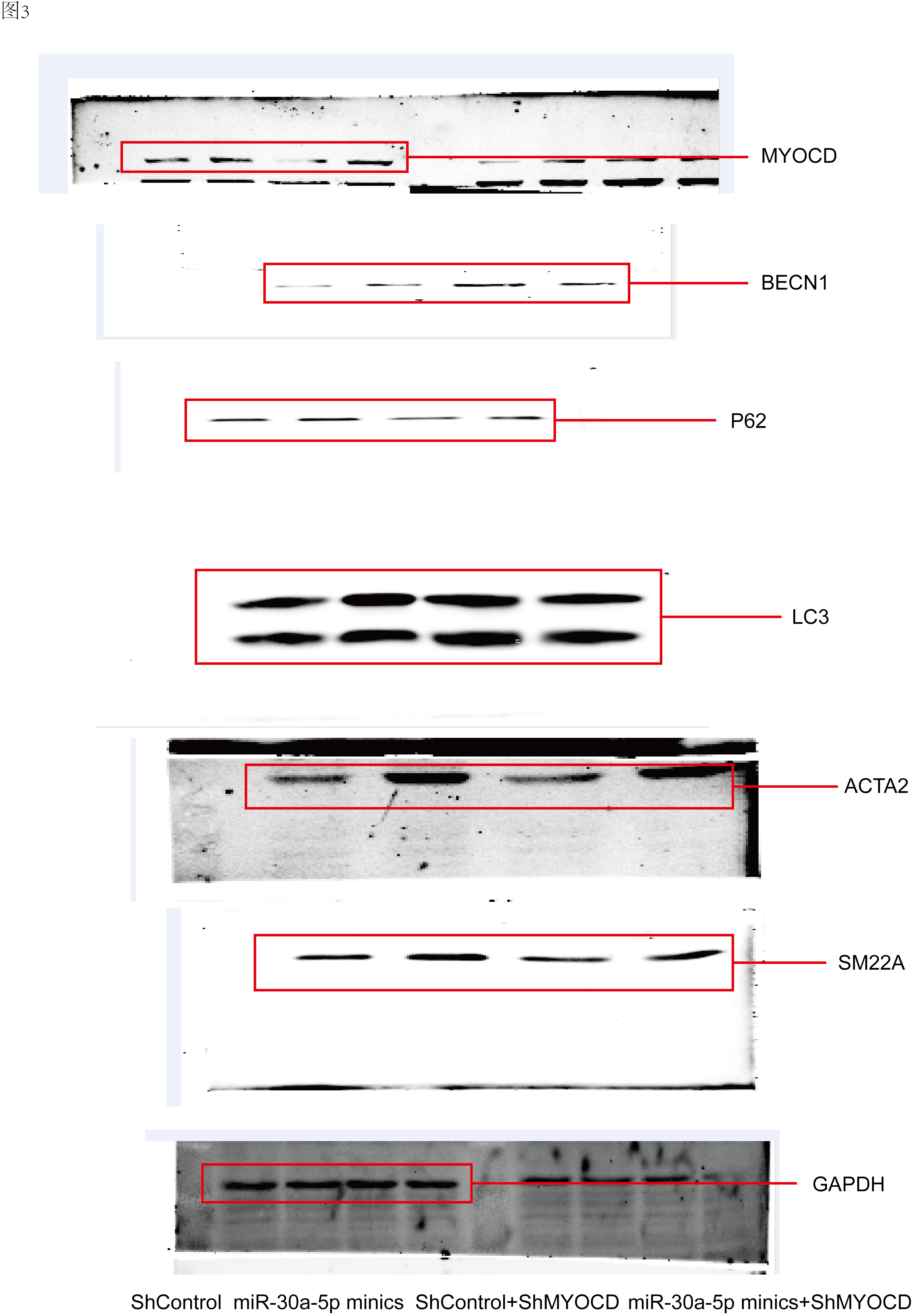

Supplement: Supplementary file 3 — Original data of western blot for Figure 3 [file 41419_2022_4588_MOESM3_ESM.png]

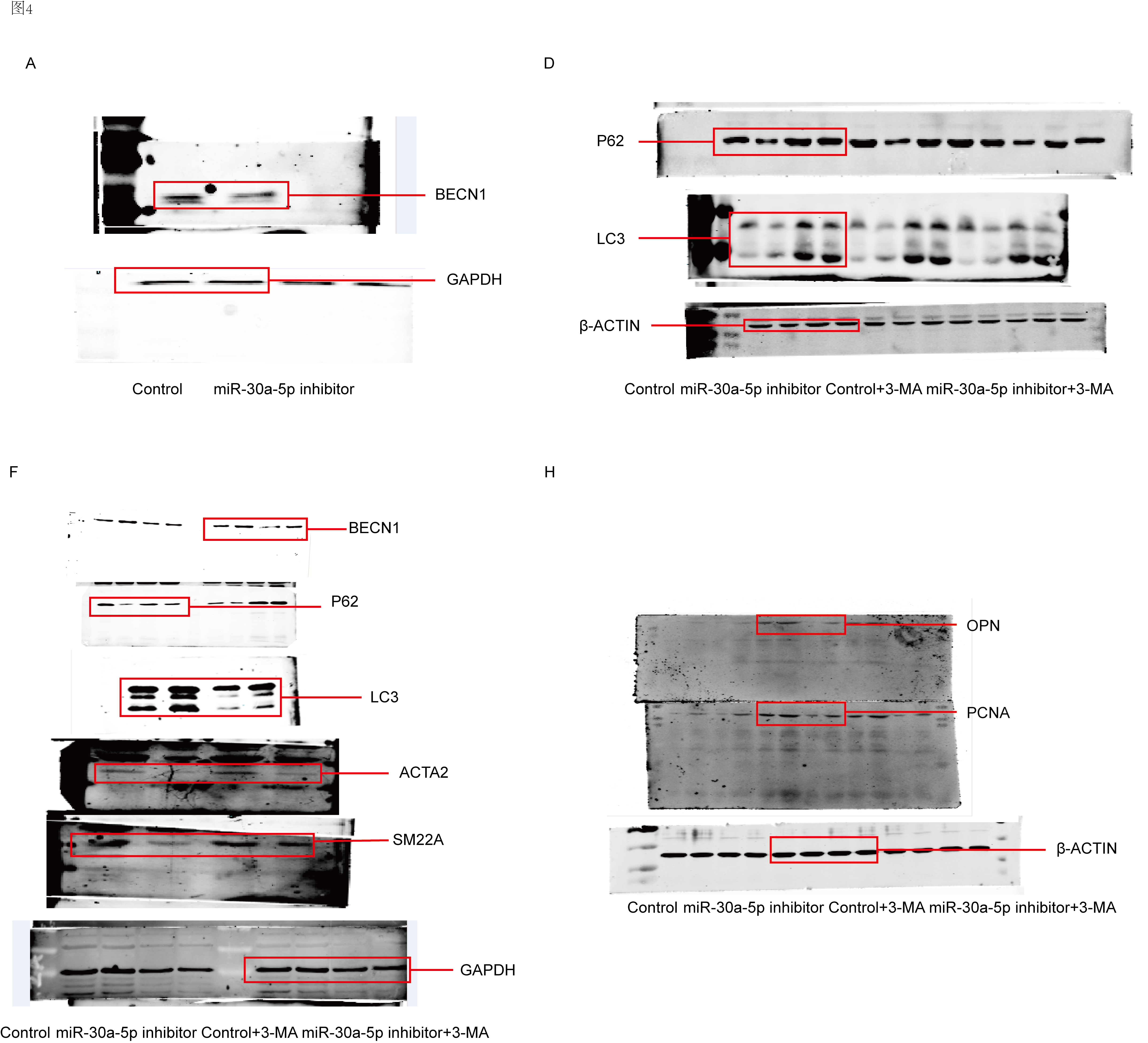

Supplement: Supplementary file 4 — Original data of western blot for Figure 4 [file 41419_2022_4588_MOESM4_ESM.png]

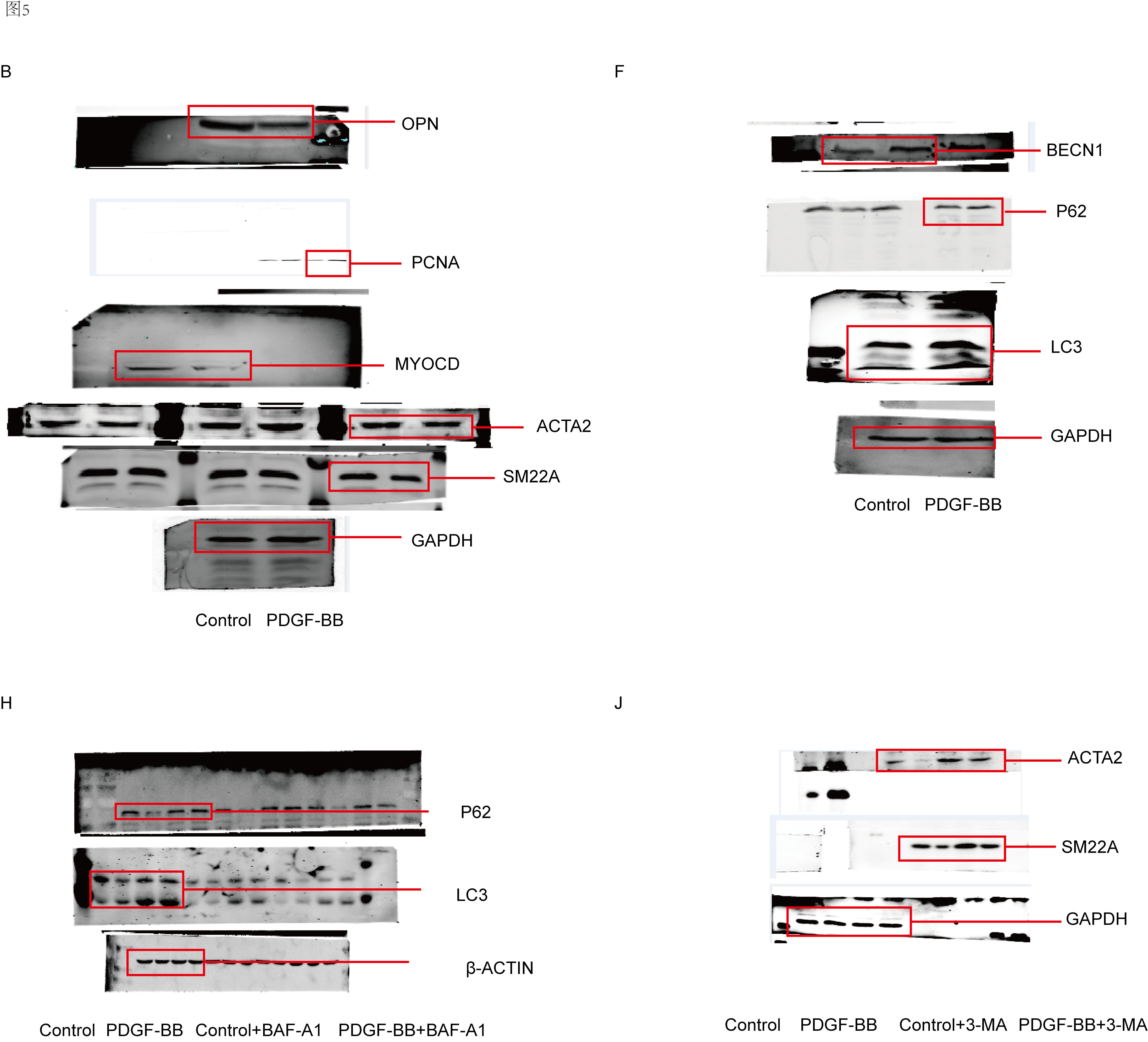

Supplement: Supplementary file 5 — Original data of western blot for Figure 5 [file 41419_2022_4588_MOESM5_ESM.png]

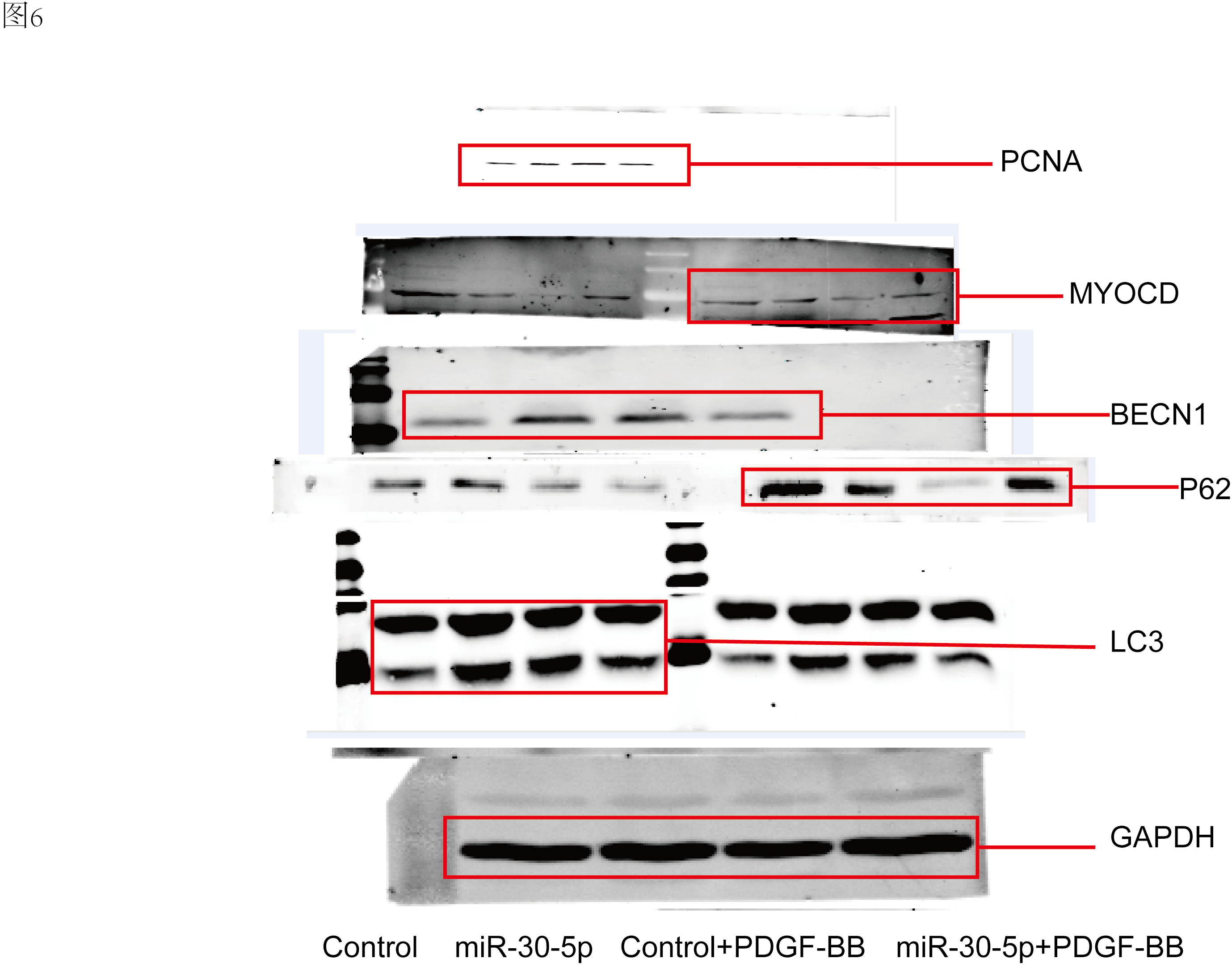

Supplement: Supplementary file 6 — Original data of western blot for Figure 6 [file 41419_2022_4588_MOESM6_ESM.png]
